# Experimental Investigation on Micro Milling of Polyester/Halloysite Nano-Clay Nanocomposites

**DOI:** 10.3390/nano9070917

**Published:** 2019-06-26

**Authors:** Guoyu Fu, Dehong Huo, Islam Shyha, Ketan Pancholi, Mohd Shahneel Saharudin

**Affiliations:** 1 School of Engineering, Newcastle University, Newcastle upon Tyne NE1 7RU, UK; 2 Department of Mechanical and Construction Engineering, Northumbria University at Newcastle, Newcastle upon Tyne NE1 8ST, UK; 3 School of Engineering, Robert Gordon University, Aberdeen AB10 7AQ, UK; 4 University Kuala Lumpur Institute of Product Design and Manufacturing (UniKL IPROM), 56100 Cheras, Kuala Lumpur, Malaysia

**Keywords:** polymeric nanocomposites, polyester, halloysite nano clay, size effect, micro-milling

## Abstract

Efficient machining of the polyester nanocomposite components requires a better understanding of machinability characteristics of such material, which has become an urgent requirement for modern industrial production. In this research, the micro-milling of polyester/halloysite nano-clay (0.1, 0.3, 0.7, 1.0 wt%) nanocomposites were carried out and the outcomes in terms of tool wear, cutting force, the size effect, surface morphology, and surface roughness were compared with those for plain polyester. In order to accomplish the machining of the material in ductile mode, the required feed per tooth was found to be below 0.3 µm. The degree of surface breakage was also found to decrease in ductile mode. A maximum flank wear VB of 0.012 mm after removing 196 mm^3^ of workpiece material was measured.

## 1. Introduction

Over the past decade, the polymer/halloysite nano clay nanocomposites have been shown to exhibit significant enhanced physical and mechanical properties, including gas barrier [1,2], flammability resistance [3,4], and ablation performance [5]. However, the use of halloysite nano clay as a nano filler in polyesters is an attractive proposition, since polyesters are inherently weak materials despite their low cost. Polyester is also the most versatile commonly available thermoset polymer and its use in preparing nanocomposite is cost-effective and adaptable to many applications. However, polyesters are brittle and highly flammable, which limits their use in many industrial applications [6,7,8]. In order to expand the range of polyester applications, the material strength of polyester must be improved either by structural modification or by adding nanoparticles. After many years of research in the area of clay-derived nano fillers, the halloysite nano clays have emerged as ideal fillers for the polyester. Earlier, it has been demonstrated that the loading of clay-derived nano fillers can significantly increase the flammability and thermal stability of the polymer matrix [9,10,11]. Adding clay-derived nano fillers are also shown to improve mechanical properties, such as fatigue resistance, fracture toughness, tensile strength, abrasion resistance, and coefficient of friction [12,13,14]. Clay-derived nano fillers including SiC, Si_3_N_4_, SiO_2_, CaCO_3_, and Al_2_O_3_, have already successfully been added to the polymer [14,15,16] to form many new polymer/nano fillers nanocomposites. The combination of halloysite nano clay and polyesters have received increasing attention in the research community and industries due to numerous benefits.

Halloysite nano clay stands out not only for its low cost, low toxicity, and high stiffness, but also due to its ability to improve the machinability and thermal stability of nanocomposites [17]. Its many benefits mean that halloysite nano clay has the potential to replace expensive nanofillers such as carbon nanotube in developing high-performance polymers and multi-functional nanocomposites [18,19]. Sharudin et al. [20] have demonstrated that the polymer/halloysite nanocomposites have better flexural properties and surface roughness in comparison to plain polymer. In another study, Saharudin et al. [21] proved that increasing the content of halloysite nano clay in polymer nanocomposite increases its resistance to environmental stress cracking and improves its storage modulus as well. Hence, halloysite nano clay is considered to be a potential enhancer of the material properties of polyester from both economic and performance perspectives [22].

Due to wide application and the excellent material properties of polymeric nanocomposites, the machinability of polyester/halloysite nano clay nanocomposites have become an urgent requirement for modern industrial production [23,24,25,26,27]. Most nanocomposites were processed by using a lithography-based technique. However, this manufacturing process is time consuming and costly [28,29]. Hence, alternative methods are much needed to replace the lithography-based method [30]. Among these, micro machining is considered as an important method to shape nanocomposites. There are four reasons. First, it is easy to achieve high machining accuracy. Second, it is easy to reveal the effect of particle content on cutting performance. Third, it has high cost effective for small scale prototyping. Last, it has the character of low industrial production costs [31]. Therefore, micromachining methods are used to understand the properties of polyester/halloysite nano clay processing and to provide data for future industrial applications.

**Figure 1:**
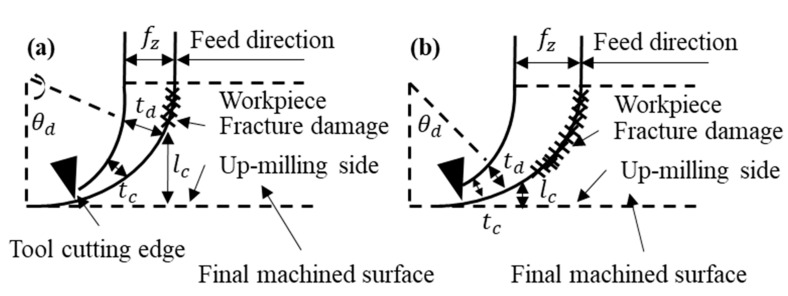
Relationship between feed rate, $f_{Z}f_{Z}$, and subsurface damage depth, $l_{c}l_{c}$, during up-milling for (**a**) low feed rate and (**b**) high feed rate.

In relation to the machinability of polymeric composites, since polyester is a brittle material [32], ductile mode is utilized to evaluate the micro milling process of the composites. Machining-induced defects caused by cutting processing, and plastic deformation inhibit machining-induced defects by controlling cutting parameters [33]. Figure 1 shows the relationship between the feed rate, $f_{Z}f_{Z}$, and subsurface damage depth, $l_{c}l_{c}$, during up-milling. As the tool cutting edge exceeds the ductile to brittle transition chip thickness, fractures will occur along the cutting shoulder. As the feed rate decreases, the subsurface damage depth, $l_{c}l_{c}$, will be longer as the brittle transition chip thickness, $t_{d}t_{d}$, will happen at the cut shoulder the upper milling side. Higher feed rate will force the fracture to be generated closer to the final machined surface.

## 2. Experimental Setup

### 2.1. Workpiece Material Preparation

Polyester/halloysite nano clay nanocomposite samples were prepared at Northumbria University, Newcastle upon Tyne, UK. An unsaturated Polyester resin (NORSODYNE O12335 AL) was obtained from the East Coast Glass Fiber (Southshields, UK). Halloysite nano clay (Al_2_Si_2_O_5_(OH)_4_) was used as a reinforcement filler and acquired from Sigma Aldrich (Irvine, UK). It had a tube-like morphology with a density of 2.53 g/cm^3^ and a surface area 64 m^2^/g. Halloysite has low electrical, thermal conductivities and strong hydrogen interactions. Its tubular morphology, high aspect ratio, and low percolation make halloysite a prospective reinforcement for polyester and other polymers.

**Figure 2:**
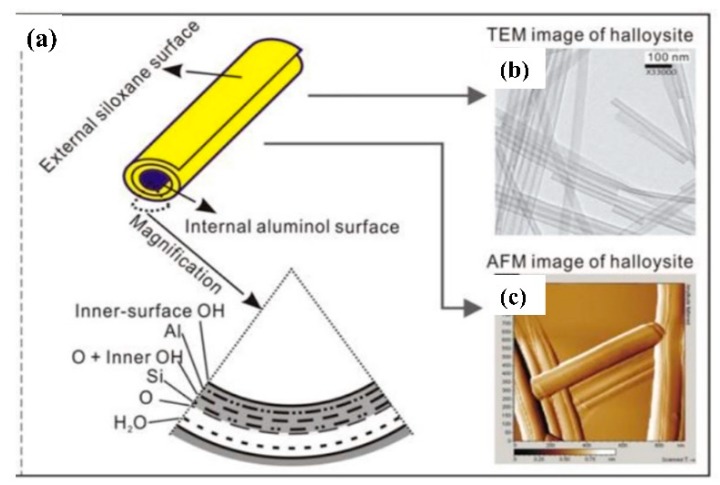
Diagram of halloysite crystalline structure (**a**) TEM images of halloysite (**b**) and Atonic Force Microscopy (AFM) image of halloysite (**c**). Reproduced from Yuan (2015) [35].

**Figure 3:**
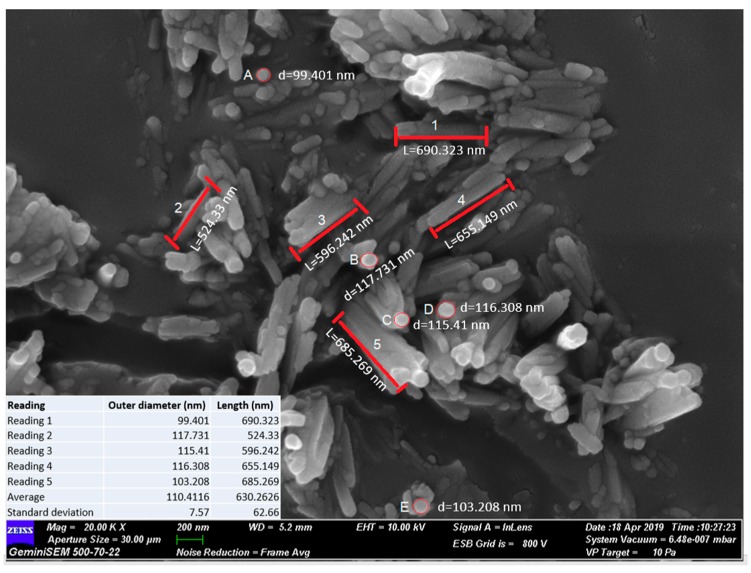
Field Emission Scanning Electron Microscope (FESEM) image of halloysite nano clay showing dimensions.

Although the typical diameter of nano clay particles is between 30 to 70 nm and the length was in range of 1–4 µm [34], as shown in Figure 2, the exact dimensions of nano clay particles used in this research were measured using ImageJ software, utilizing the ratio of pixel and length relationship. From the image processing analysis, the average outer diameter was found to be 110.41 nm ± 7.6 nm, and the average length is 630.26 nm ± 62.7 nm. It was technically challenging to obtain information in relation to the inner diameter of the holloysite nano clay, Figure 3. However, a study by Levis and Deasy have reported that the value of inner diameter for similar halloysite is about 100 nm.

**Figure 4:**
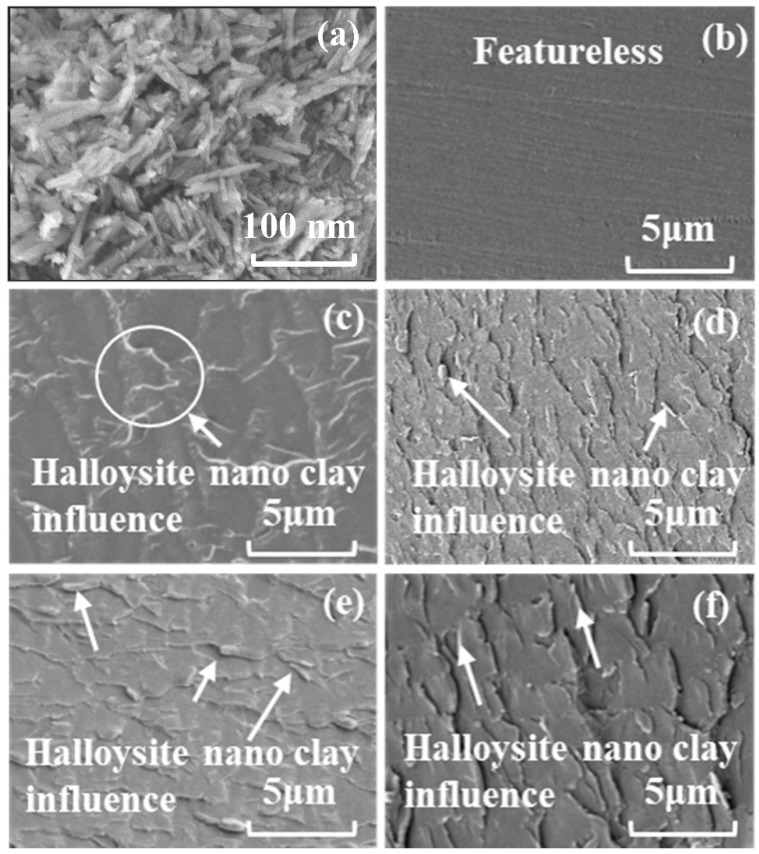
SEM image of materials surface and nano fillers: (**a**) Halloysite nano clay, (**b**) plain polyester, (**c**) halloysite nano clay 0.1 wt%/polyester, (**d**) halloysite nano clay 0.3 wt%/polyester, (**e**) halloysite nano clay 0.7 wt%/polyester, (**f**) halloysite nano clay 1.0 wt%/polyester. Reproduced from Saharudin (2017) [36].

**Figure 5:**
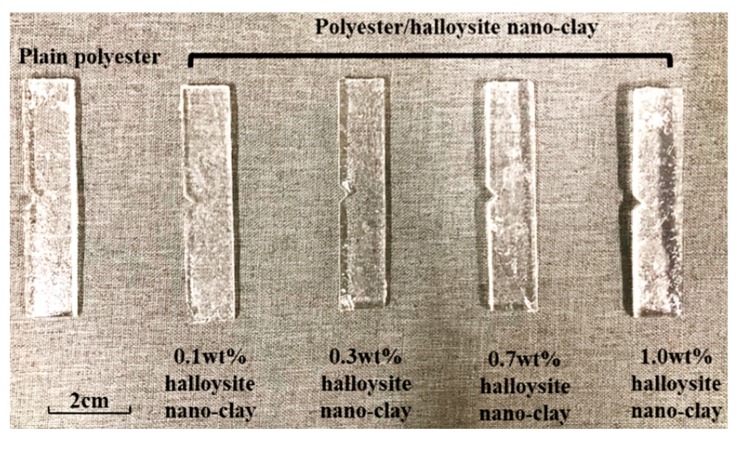
Workpiece prepared for micro milling experimentation.

The manual mixing and vacuum degassing of polyester resin and nano clay was carried out for 10 min before pouring into a mold. The composites mixture was poured into a silicone mold and cured at room temperature for 24 h, followed by post-curing at temperature of 60 °C in an oven for 2 h to achieve complete crosslinking. The images in Figure 4c–f show the influence of halloysite nano clay in the polyester matrix. It can be observed that no agglomerates were visible, and therefore, the halloysite nano clay particles were homogenously dispersed. Moreover, the maximum clay reinforcement in this research was 1.0 wt% since this is optimal clay reinforcement as reported in our previous work [22]. Dispersing higher clay fraction is also challenging and agglomerations of halloysite nano clays are likely to form, as stress raiser will cause the mechanical properties of composites to deteriorate. Images of the prepared polyester/halloysite nano clay nanocomposite samples with different nano filler contents of 0.1, 0.3, 0.7, and 1.0 wt% as well as plain polyester are shown in Figure 5.

### 2.2. Machine Setup

**Figure 6:**
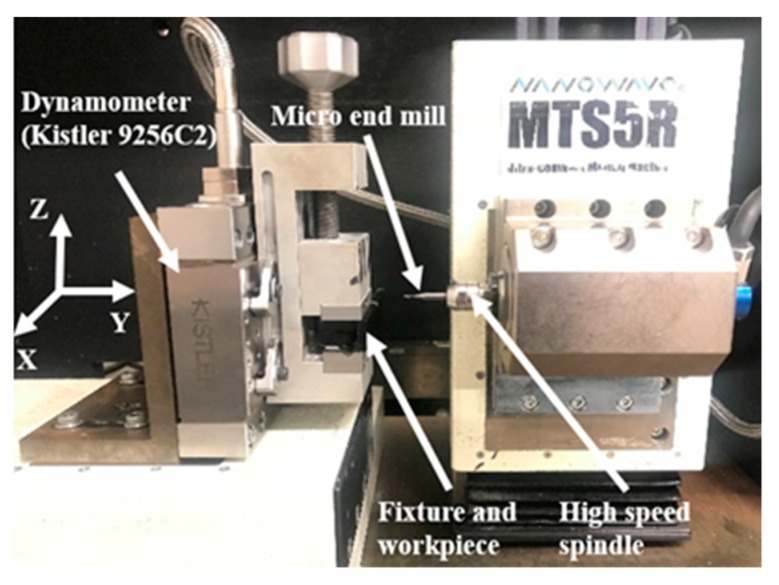
Setup for the milling process and cutting force measurement.

In the experiment, dry machining was used without air cooling, as shown in Figure 6. Each workpiece was placed on the machine platform (MTS5R), and machine platform spindle was used for machining the workpiece. The range of spindle speeds for this machine is from 5000 to 80,000 rpm. The machining platform can be used for high feed rate and high cutting speed working condition; meanwhile, it can achieve the milling condition of a low feed rate. Its minimum feed rate is 0.1 μm/rev for the X-, Y-, and Z axes. Meanwhile, the X- and Y-axis are the workpiece rectangular surface, which is the plane at the beginning of the milling process. The cutting force parameter was measured by Kistler dynamometer piezoelectric device (9256C2), which was set up to record the forces from X-, Y-, and Z-axes, instantly.

### 2.3. Micro-End-Mill

**Figure 7:**
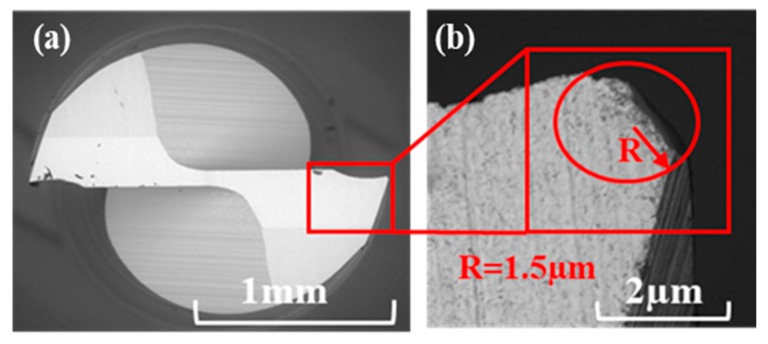
Uncoated tungsten carbide micro end mill (1 mm) with cutting edge radius measurement: (**a**) Top view of tool, (**b**) cutting edge radius measurement method.

Uncoated two-flutes micro-end tools with 1 mm nominal diameter were used in this research. Additional tool specifications are shown in Table 1. The cutting-edge radius for the new tool was 1.5 µm, which was measured using SEM as shown in Figure 7.

### 2.4. Micro Milling Conditions

Two sets of micro milling experiments have been used to gain an understanding of the suitability for machining of the nanocomposites. One set was designed to determine the effect of content on the surface morphology, and the purpose of the other set was to quantify the size effect. The aim of the first experiment is to study the milling process through three level (5, 10, and 15 µm/rev) of feed per tooth and 6 levels (15.7, 31.4, 62.8, 94.2, 125.6, 188.4 m/min) of cutting speed. The cutting conditions in the first set of experiments are shown in Table 2. The parameters of this set combined those in the machinability study of polymer/GNP from Arora et al. [36] and the machinability study of Mg/nanofiller from Teng et al. [37].

The aim of the second set of experiments was to determine the size effect and minimum chip thickness (MCT) of nanocomposites, and the milling conditions detailed in Table 3. The parameters of this set were taken from the size study of Mg/nano filler from Teng et al. [37]. Each test was repeated twice so that the accuracy of the experimental results could be ensured. The milling depth in both experiments was 0.1 mm. The dimensions of the specimens used for these tests were 14 mm × 70 mm × 3 mm.

### 2.5. Characterization Methods

#### 2.5.1. Surface Roughness Measurement

An ultrasonic bath was used to clean the machined samples before surface measure. A portable surface roughness measurement instrument (Mitutoyo Talysurf SJ-310) was used to measure the surface roughness of the bottom machined slot surface. Five measurements were taken from different points to ensure measurement accuracy [33]. Surface morphology was also analyzed using a scanning electron microscope (Hitachi TM3030). Upon machining, the evident appearance of chip adhesion on the tool was analyzed using energy dispersive X-ray spectroscopy (EDX).

#### 2.5.2. Tool Wear Measurement

Tool wear is a very important factor that can influence the surface roughness, surface morphology, and burr formation [38,39,40,41,42,43,44]. Previous studies have shown that the main source of error in a final milled micropart is tool deflection due to tool wear [38,44]. In this experiment, we measured the flank wear of the tool. In order to quantify the effect of nano filler loading on tool wear, a separate set of machining tests were conducted. The tool was first characterized under a SEM microscope by observing its initial condition. The tool run consisted of 150 13 mm long half-immersion cut and remove 196 mm^3^ volume of polyester/halloysite nano clay 1.0 wt% nanocomposites. The volume removed for every slot was 1.3 mm^3^ (1 mm × 13 mm × 0.1 mm). The axial depth-of-cut, FPT, and spindle speed were maintained at 0.1 mm, 5 μm, and 40,000 rpm, respectively, for the duration of each runs. At the end of each run, the tool tip was imaged under the SEM to record the extent of wear.

## 3. Results and Discussion

### 3.1. Surface Roughness

**Figure 8:**
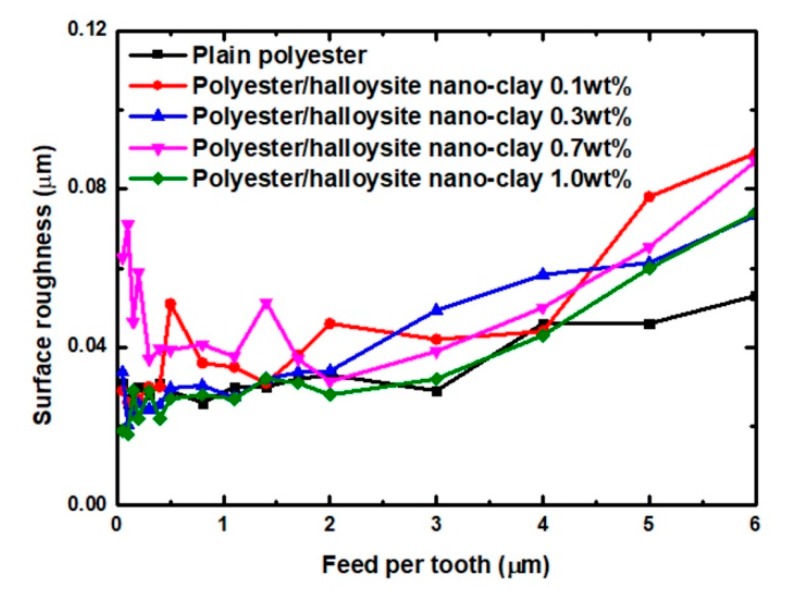
Effect of feed per tooth on the surface roughness.

Figure 8 shows the relationship between feed per tooth and surface roughness of polyester/halloysite nano clay nanocomposites. It shows that the addition of halloysite nano clay led to a slight increase in surface roughness. This may be due to the fracture toughness increase with the GNP content increase [42]. Meanwhile, the addition of nano clay did not change the machining character of brittle material of polymer that surface roughness increased with feed per tooth (FPT) value increase [36]. This is due to the limited effect of halloysite nano clay on improving fracture toughness [43]. If the nanoparticle can significantly improve the fracture toughness [43,45], the surface roughness of polymer composites will first decrease and then increase as the value of FPT increase. The increase of fracture toughness is too small to cause a change in the facture mode [43]. Hence, polyester/halloysite nano clay still has machining character of brittle material.

Meanwhile, the surface roughness increased with the FPT. This is because the fracture crack tip is affected by normal tangential load when the tool is in contact, with the significant concentrations of residual tensile and shear stresses are formed around the cutting area at high feed per tooth. This can cause internal micro-cracks when machining at higher feed rates [46]. In order to achieve good surface quality, the value of FPT should be kept below 2 µm.

### 3.2. Cutting Force Analysis

Determining the resulting cutting force and specific cutting energy were the two main methods used in the analysis of the machinability of brittle material. Specific cutting energy is the energy taken to remove a unit volume of the material and can be used as an indicator in evaluate the size effect. Using the computed thrust force, $F_{t}F_{t}$, and the cutting force, $F_{c}F_{c}$, specific cutting energy, U, is calculated using the trapeze integration method shown in Equation (1) [47].

(1) $$U=\frac{V_{c}}{V_{rem}}\times \int_{0}^{T_{c}}\sqrt{F_{t}^{2}+F_{c}^{2}}dt$$

where $V_{c}V_{c}$ and $V_{\mathrm{rem}}V_{\mathrm{rem}}$ are the cutting speed (m/min) and the material removal volume (mm^3^), while $T_{c}T_{c}$ is the cutting time (seconds).

**Figure 9:**
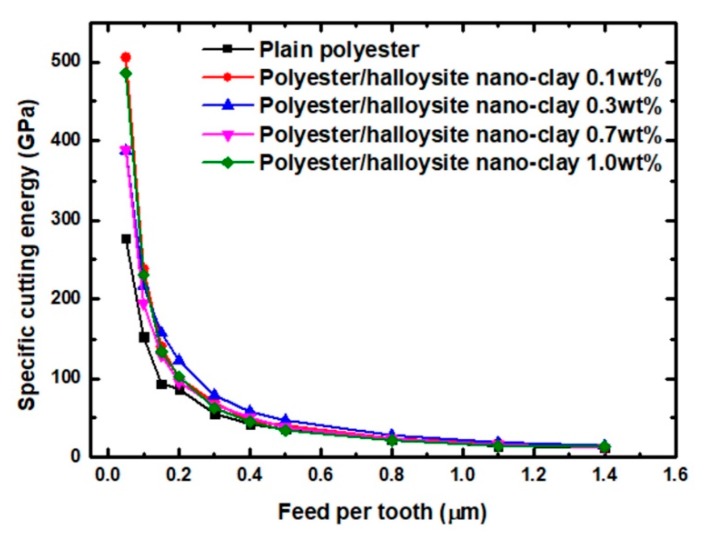
Experimental specific cutting energy for polyester/halloysite nano clay with the second set of machining parameters.

Figure 9 shows the specific cutting energy of various polyester/halloysite nano clays. Regardless of the machining parameters, higher specific cutting energy reflecting the dominance of ductile mode machining were observed at low feed per tooth (≤0.3 µm) [33]. When the feed per tooth exceeded 0.5 µm, the facture mode gradually developed to brittle fracture. Hence, the transition between ductile to brittle machining mode occurred between 0.3 to 0.5 µm. When the feed per tooth was below 0.3 µm, the higher specific cutting energy can be attributed to the shearing of material by the tool cutting edge along a defect-free plane. Meanwhile, the extremely high specific cutting energy at the FPT of 0.05 µm could be explained by the potential dominance of ploughing. The interfacial friction between the tool flank face and newly machined surfaces caused the extremely highly specific cutting energy. When the feed per tooth was over 0.5 µm, the specific cutting energy became relatively stable [33]. In order to achieve machined surfaces without any surface defects, the FPT should be below 0.3 μm under the ductile mode. Due to the addition of nanoparticles, the specific cutting energy of polyester/halloysite nano clay had an upward trend. This may be due to three reasons. One is that the storage modulus increased with clay content increase [48,49]. Meanwhile, the highest specific cutting energy was observed by 0.1 wt% reinforcement instead of 1.0 wt%. This indicates that nano clay clusters may act as flaws, hence lowering the storage modulus from the expected level [21]. A second possibility is that the interfacial shear strength increases slightly as nano clay content increase [50]. Thirdly, the presence of nano clay may slightly increase the fracture toughness [43].

The resulting cutting energy was generated directly by the relative motion of the cutting tool with respect to the workpiece during machining. It occurred in the same direction as cutting tool movement. Using the computed thrust force, $F_{t}F_{t}$, and the cutting force, $F_{c}F_{c}$, specific cutting energy, F, calculate using the method shown in Equation (2) [51].

(2) $$F=\sqrt{F_{t}^{2}+F_{c}^{2}}$$

**Figure 10:**
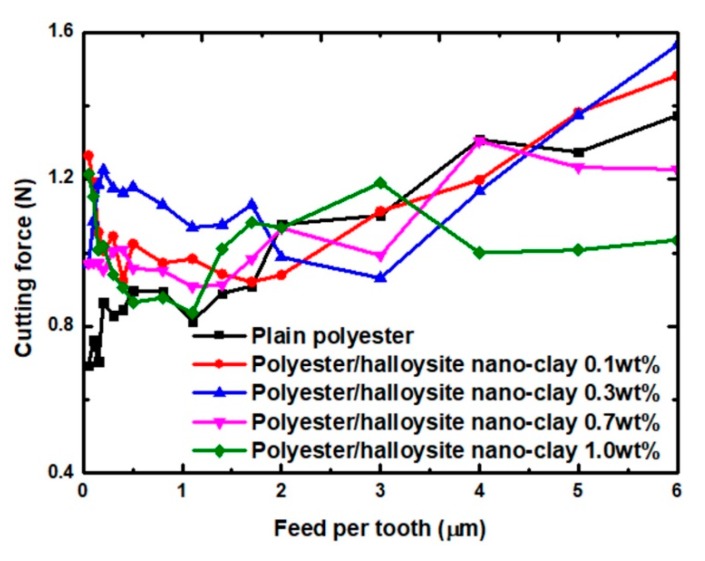
Effect of feed per tooth (FPT) value on cutting force.

Figure 10 presents the resulting cutting force of various nanocomposites with different values of feed per tooth. The addition of particles did not cause significant differences in the cutting forces of polyester/halloysite nano clay and plain polyester. The cutting force trend of polyester/halloysite nano clay nanocomposites rises first (≤2.0 µm) and then decreases with the FPT increase. The first variation area is that the cutting force decreased as the feed per tooth increased. The reason for the reduction in cutting force is that plastic deformation and elastic deformation coexist in this region. The second variation area is that the cutting force increased as the feed per tooth increases as the feed per tooth increases. Meanwhile, chaotic forces and chattering were observed at high feed rates. This can result in uneven stress loading within the cutting zone, thereby promoting the growth of microcracks.

### 3.3. Surface Morphology

**Figure 11:**
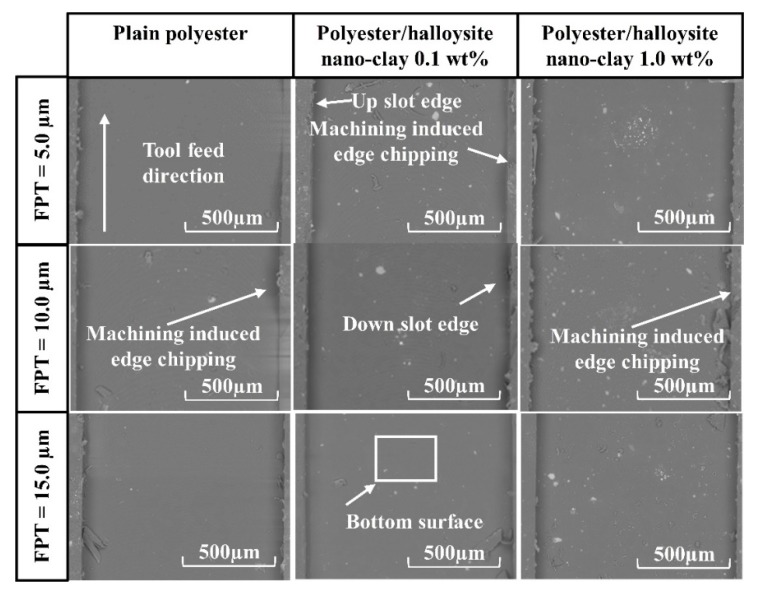
SEM graphs of typical machined slots at FPT = 5.0, 10.0, and 15.0 µm.

**Figure 12:**
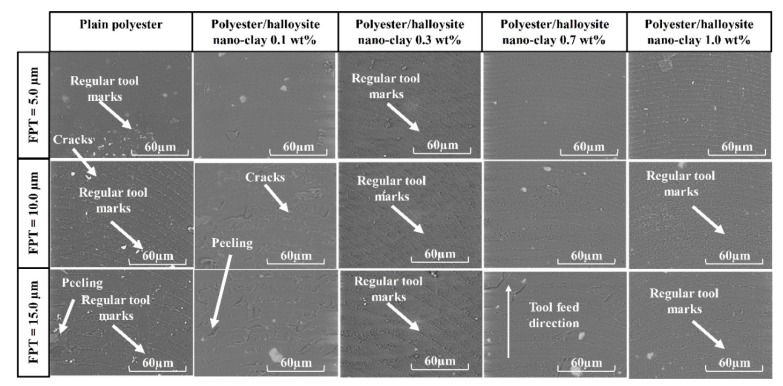
Bottom surface of machined slot at FPT = 5.0, 10.0, and 15.0 µm (600×).

**Figure 13:**
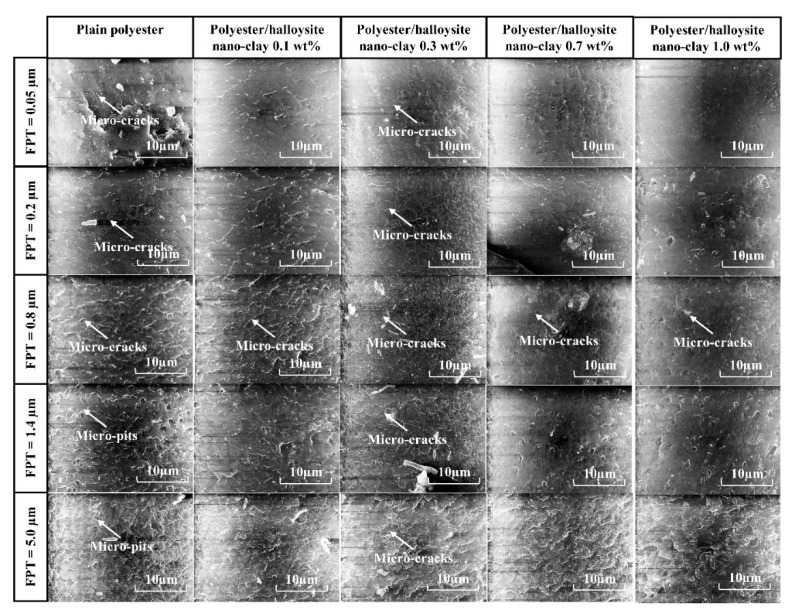
SEM graphs of slot bottoms of various polyester/halloysite nano clay at zoom 6000×.

For brittle materials, when the FPT was less than the minimum cutting thickness (MCT), ploughing occurred, and the ploughing process caused the complex deformation of the machined surface. Therefore, it is necessary to select appropriately milling conditions, such as cutting speed and feed rate, in order to understand the factors influencing surface formation in polyester/halloysite nano clay. Figure 11 shows SEM graphs of typical machined slots. Here, different polyester/halloysite nano clay at FPT = 5.0, 10.0, and 15.0 µm showed no significant differences in machined slots. In order to more thoroughly understand the effect of nano clay on the surface morphology, images of the bottom machined surface were collected at zoom = 600× and 6000×, as shown in Figure 12 and Figure 13. Figure 12 shows that tool marks are clearly present on the polyester/halloysite nano clay composites. However, tool marks do not significantly differ with various particle contents. This is because fracture toughness did not significantly influence the halloysite nano clay content [43]. Figure 12 shows SEM graphs of the slot bottom of various polyester/halloysite nano clay by zoom 6000×. As for plain polyester, with the increase of FPT, the trend of surface morphology changed. There are many microcracks on the machined surface on the small FPT value. With the increase of FPT, many small micro-pits appear on the surface. This is due to the fracture mode changing from ductile to brittle mode with the increase in FPT [33]. With increasing halloysite nano clay content, micro-crack, and micro-pit frequency was reduced. There are two reasons for this: Firstly, the addition of halloysite nano clay lowers the stress crack resistance [21], and secondly, the fracture toughness increases slightly.

### 3.4. Tool Wear

**Figure 14:**
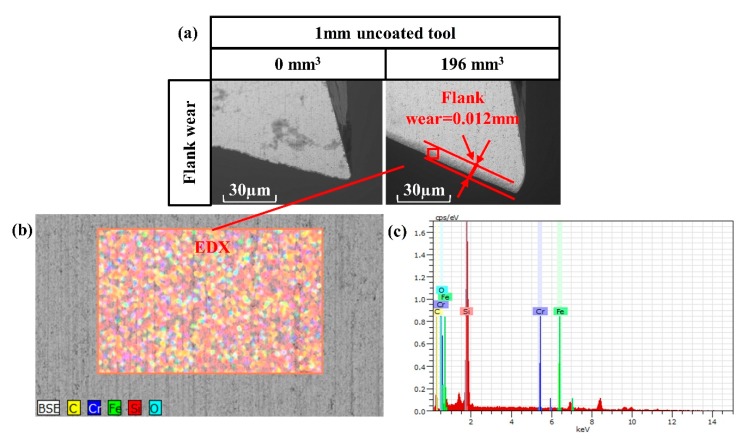
Tool wear: (**a**) SEM micrographs of the tool wear; (**b**,**c**) energy dispersive X-ray spectroscopy (EDX) analysis of the cutting edge.

Figure 14 shows the 1 mm uncoated tool characterized by both SEM and EDX after removal of 196 mm^3^ of polyester/halloysite nano clay 1.0 wt% nanocomposites at cutting conditions of 40,000 rpm spindle speed and 5 µm FPT. The chip adhesion on the tool surface may be related to generation of heat in the cutting zone during machining [52]. The kinetic energy supplied by the compressive motion from a negative effective rake angled tool is converted into heat during the material removal process due to plastic deformation. The high temperature generated within the cutting zone may increase the diffusion rate and chemical interactions between the cutting tool and the polyester nanocomposites workpiece [33]. Figure 12 shows nanocomposites adhered onto the rake face of the tool. The flank wear of the tool was considered to have undergone a series of effects, from thermal to the tribo-chemical reaction. It consisted of surface oxidation (also observed by the EDX analysis in Figure 8) and the formation of polymer oxidation-like particles that adhered to the uncoated tool. In addition, the crater wear did not occur in this study. The resulting flank wear V_B_ was 0.012 mm. This value is very small compared to metal matrix nanocomposites [53].

## 4. Conclusions

The micro-milling machining of plain polyester and polyester reinforced with halloysite nano clay (0.1, 0.3, 0.7, 1.0 wt%) was investigated using 1 mm uncoated tools. The study encompassed evaluations of nano filler content and cutting conditions on surface topography, surface roughness, and cutting force. The following conclusions can be drawn:

- As the nanoparticle content increases, surface roughness of the machined surfaces increases. In order to achieve good surface quality, the FPT value should be below 2 µm.
- The machined surfaces exhibit uniform machining marks when machining at small values of feed per tooth (≤0.3 μm) under the ductile mode. Due to the addition of nanoparticles, the specific cutting energy of polyester/halloysite nano clay has an upward trend. This is due to the storage modulus increasing with clay loading, and the fact that the interfacial shear strength increased slightly with higher nano clay loading.
- A maximum flank wear VB of 0.012 mm was measured after removing 196 mm^3^ from the workpiece.

## Figures and Tables

**Table 1:** Micro-end milling specifications.

Properties	Value
Tool diameter	1 mm
Number of Flutes	2
Helix Angle	30°
Tool edge radius	1.5 µm

**Table 2:** Cutting conditions used in the experiments.

Cutting Parameters	Level 1	Level 2	Level 3	Level 4	Level 5	Level 6
Feed per tooth (µm)	5	10	15	/	/	/
Feed rate (µm/tooth)	10	20	30	/	/	/
Cutting speed (m/min)	15.7	31.4	62.8	94.2	125.6	188.4
Spindle speed (rpm)	5000	10,000	20,000	30,000	40,000	60,000
Depth of cut (µm)	100	/	/	/	/	/

**Table 3:** Cutting conditions for the size effect experiment.

Feed per Tooth (µm/tooth)	Cutting Speed (m/min)	Spindle Speed (rpm)	Depth of Cut (µm)
0.05, 0.1, 0.15, 0.2, 0.3, 0.4, 0.5, 0.8, 1.1, 1.4, 1.7, 2.0, 3.0, 4.0, 5.0, 6.0	62.8	40,000	100

## References

[1] Yano K., Usuki A., Okada A., Kurauchi T., Kamigaito O. (1993). Synthesis and properties of polyimide–clay hybrid. J. Polym. Sci. Part A Polym. Chem..

[2] Messersmith P.B., Giannelis E.P. (1995). Synthesis and barrier properties of poly(epsilon-caprolactone)-layered silicate nanocomposites. J. Polym. Sci. Part A Polym. Chem..

[3] Gilman J.W. (1999). Flammability and thermal stability studies of polymer layered-silicate (clay) nanocomposites. Appl. Clay Sci..

[4] Rybiński P., Janowska G. (2013). Influence synergetic effect of halloysite nanotubes and halogen-free flame-retardants on properties nitrile rubber composites. Thermochim. Acta.

[5] Vaia R. (1999). Polymer/layered silicate nanocomposites as high performance ablative materials. Appl. Clay Sci..

[6] Bonnia N.N. (2010). Mechanical properties and environmental stress cracking resistance of rubber toughened polyester/kenaf composite. Express Polym. Lett..

[7] Albdiry M.T., Yousif B.F., Ku H. Fracture toughness and toughening mechanisms of unsaturated polyester-based clay nanocomposites. Proceedings of the 13th International Conference on Fracture.

[8] Attia N.F., Hassan M.A., Nour M.A., Geckeler K.E. (2014). Flame-retardant materials: Synergistic effect of halloysite nanotubes on the flammability properties of acrylonitrile-butadiene-styrene composites. Polym. Int..

[9] Sumita M., Shizuma T., Miyasaka K., Ishikawa K. (1983). Effect of reducible properties of temperature, rate of strain, and filler content on the tensile yield stress of nylon 6 composites filled with ultrafine particles. J. Macromol. Sci. Part B.

[10] Kuo M.C., Tsai C.M., Huang J.C., Chen M. (2005). PEEK composites reinforced by nano-sized SiO2 and A1 2O3^Os particulates. Mater. Chem. Phys..

[11] Attwood T., Dawson P., Freeman J., Hoy L., Rose J., Staniland P. (1981). Synthesis and properties of polyaryletherketones. Polymer.

[12] Goyal R., Negi Y., Tiwari A. (2005). Preparation of high performance composites based on aluminum nitride/poly(ether–ether–ketone) and their properties. Eur. Polym. J..

[13] Cassagnau P. (2003). Payne effect and shear elasticity of silica-filled polymers in concentrated solutions and in molten state. Polymer.

[14] Aly A.A., Zeidan E.-S.B., Alshennawy A.A., El-Masry A.A., Wasel W.A. (2012). Friction and Wear of Polymer Composites Filled by Nano-Particles: A Review. World J. Nano Sci. Eng..

[15] Wang Q.-H., Xue Q.-J., Liu W.-M., Chen J.-M. (2000). The friction and wear characteristics of nanometer SiC and polytetrafluoroethylene filled polyetheretherketone. Wear.

[16] Yamamoto I., Higashihara T., Kobayashi T. (2003). Effect of Silica-Particle Characteristics on Impact/Usual Fatigue Properties and Evaluation of Mechanical Characteristics of Silica-Particle Epoxy Resins. JSME Int. J. Ser. A.

[17] Du M., Guo B., Jia D. (2010). Newly emerging applications of halloysite nanotubes: A review. Polym. Int..

[18] Lecouvet B., Horion J., D’Haese C., Bailly C., Nysten B. (2013). Elastic modulus of halloysite nanotubes. Nanotechnology.

[19] Lu D., Chen H., Wu J., Chan C.M. (2011). Direct Measurements of the Young’s Modulus of a Single Halloysite Nanotube Using a Transmission Electron Microscope with a Bending Stage. J. Nanosci. Nanotechnol..

[20] Saharudin M.S., Wei J., Shyha I., Inam F. (2017). Flexural Properties of Halloysite Nanotubes- Polyester Nanocomposites Exposed to Aggressive Environment. Int. J. Chem. Mol. Nucl. Mater. Metall. Eng..

[21] Saharudin M.S., Wei J., Shyha I., Inam F. (2017). Environmental Stress Cracking Resistance of Halloysite Nanoclay-Polyester Nanocomposites. World J. Eng. Technol..

[22] Saharudin M.S., Atif R., Shyha I., Inam F. (2017). The degradation of mechanical properties in halloysite nanoclay–polyester nanocomposites exposed to diluted methanol. J. Compos. Mater..

[23] Luo B.-H., Hsu C.-E., Li J.-H., Zhao L.-F., Liu M.-X., Wang X.-Y., Zhou C.-R. (2013). Nano-Composite of Poly(L-Lactide) and Halloysite Nanotubes Surface-Grafted with L-Lactide Oligomer Under Microwave Irradiation. J. Biomed. Nanotechnol..

[24] Wei W., Abdullayev E., Hollister A., Mills D., Lvov Y.M. (2012). Clay Nanotube/Poly (methyl methacrylate) Bone Cement Composites with Sustained Antibiotic Release. Macromol. Mater. Eng..

[25] Cavallaro G., Donato D.I., Lazzara G., Milioto S. (2011). Films of Halloysite Nanotubes Sandwiched between Two Layers of Biopolymer: From the Morphology to the Dielectric, Thermal, Transparency, and Wettability Properties. J. Phys. Chem. C.

[26] Yuan P., Tan D., Annabi-Bergaya F. (2015). Properties and applications of halloysite nanotubes: Recent research advances and future prospects. Appl. Clay Sci..

[27] Liu M., Jia Z., Jia D., Zhou C. (2014). Recent advance in research on halloysite nanotubes-polymer nanocomposite. Prog. Polym. Sci..

[28] Kim W.-H., Park C.S., Son J.Y. (2014). Nanoscale resistive switching memory device composed of NiO nanodot and graphene nanoribbon nanogap electrodes. Carbon.

[29] López-Suárez M., Torres F., Mestres N., Rurali R., Abadal G. (2014). Fabrication of highly regular suspended graphene nanoribbons through a one-step electron beam lithography process. Microelectron. Eng..

[30] Huang C.-H., Su C.-Y., Okada T., Li L.-J., Ho K.-I., Li P.-W., Chen I.-H., Chou C., Lai C.-S., Samukawa S. (2013). Ultra-low-edge-defect graphene nanoribbons patterned by neutral beam. Carbon.

[31] Huo D., Cheng K. (2013). Micro cutting mechanics. Micro-Cutting: Fundamentals and Applications.

[32] Zhang M., Singh R.P. (2004). Mechanical reinforcement of unsaturated polyester by AL2O3 nanoparticles. Mater. Lett..

[33] Choong Z.J., Huo D., Degenaar P., O’Neill A. (2019). Micro-machinability and edge chipping mechanism studies on diamond micro-milling of monocrystalline silicon. J. Manuf. Process..

[34] Levis S., Deasy P. (2002). Characterisation of halloysite for use as a microtubular drug delivery system. Int. J. Pharm..

[35] Saharudin M. (2017). Mechanical Properties of Polyester Nano-Composites Exposed to Liquid Media. Ph.D. Thesis.

[36] Arora I., Samuel J., Koratkar N. (2013). Experimental investigation of the machinability of epoxy reinforced with graphene platelets. J. Manuf. Sci. Eng..

[37] Teng X., Huo D., Wong E., Meenashisundaram G., Gupta M., Wong W.L.E. (2016). Micro-machinability of nanoparticle-reinforced Mg-based MMCs: An experimental investigation. Int. J. Adv. Manuf. Technol..

[38] Filiz S., Conley C.M., Wasserman M.B., Ozdoganlar O.B. (2007). An experimental investigation of micro-machinability of copper 101 using tungsten carbide micro-endmills. Int. J. Mach. Tools Manuf..

[39] Lee K., Dornfeld D.A. (2005). Micro-burr formation and minimization through process control. Precis. Eng..

[40] Schaller T., Bohn L., Mayer J., Schubert K. (1999). Microstructure grooves with a width of less than 50 μm cut with ground hard metal micro end mills. Precis. Eng..

[41] Bissacco G., Hansen H.N., De Chiffre L. (2005). Micromilling of hardened tool steel for mould making applications. J. Mater. Process. Technol..

[42] Alamri H., Low I.M. (2012). Effect of water absorption on the mechanical properties of nano-filler reinforced epoxy nanocomposites. Mater. Des..

[43] Chaeichian S., Wood-Adams P.M., Hoa S.V., Wood-Adams P.M. (2015). Fracture of unsaturated polyester and the limitation of layered silicates. Polym. Eng. Sci..

[44] Samuel J., Hsia K.J., Dikshit A., Devor R.E., Kapoor S.G. (2009). Effect of Carbon Nanotube (CNT) Loading on the Thermomechanical Properties and the Machinability of CNT-Reinforced Polymer Composites. J. Manuf. Sci. Eng..

[45] Jasinevicius R.G., Andreeta M.R.B., Fossa J.S., Hernandes A.C., Duduch J.G., Demont P., Puech P. (2009). Brittle and ductile removal modes observed during diamond turning of carbon nanotube composites. Proc. Inst. Mech. Eng. Part B J. Eng. Manuf..

[46] Carr J.W., Feger C. (1993). Ultraprecision machining of polymers. Precis. Eng..

[47] Rodrigues A.R., Coelho R.T. (2007). Influence of the tool edge geometry on specific cutting energy at high-speed cutting. J. Braz. Soc. Mech. Sci. Eng..

[48] Pramoda K.P., Liu T. (2004). Effect of moisture on the dynamic mechanical relaxation of polyamide-6/clay nanocomposites. J. Polym. Sci. Part B Polym. Phys..

[49] Wu Z., Zhou C., Qi R., Zhang H. (2002). Synthesis and characterization of nylon 1012/clay nanocomposite. J. Appl. Polym. Sci..

[50] Nakamura R., Netravali A.N., Hosur M.V. (2012). Effect of halloysite nanotube incorporation in epoxy resin and carbon fiber ethylene/ammonia plasma treatment on their interfacial property. J. Adhes. Sci. Technol..

[51] Altintaş Y., Budak E. (1995). Analytical Prediction of Stability Lobes in Milling. CIRP Ann..

[52] Kumar M.N., Mahmoodi M., TabkhPaz M., Park S., Jin X. (2017). Characterization and micro end milling of graphene nano platelet and carbon nanotube filled nanocomposites. J. Mater. Process. Technol..

[53] Teng X. (2018). Investigation into micro machinability of Mg based metal matrix composites ( MMCs ) reinforced with nanoparticles. Int. J. Adv. Manuf. Technol..

